# A12 IMPACT OF CADX ON OPTICAL DIAGNOSIS OF SERRATED AND ADVANCED COLORECTAL LESIONS: A PROSPECTIVE IMPLEMENTATION STUDY

**DOI:** 10.1093/jcag/gwaf042.012

**Published:** 2026-02-13

**Authors:** M Oleksiw, D K Rex, H Pohl, C Hassan, R Djinbachian, D von Renteln

**Affiliations:** Centre Hospitalier de l’Universite de Montreal, Montreal, QC, Canada; Indiana University School of Medicine, Indianapolis, IN; Dartmouth College, Hanover, NH; IRCCS Humanitas Research Hospital, Rozzano, Lombardia, Italy; Centre Hospitalier de l’Universite de Montreal, Montreal, QC, Canada; Centre Hospitalier de l’Universite de Montreal, Montreal, QC, Canada

## Abstract

**Background:**

Computer Aided Diagnosis (CADx) can classify colorectal polyps as either neoplastic or hyperplastic during colonoscopy.

**Aims:**

We aimed to assess CADx performance and its impact on endoscopist optical diagnosis for sessile serrated lesions (SSLs), traditional serrated adenomas (TSAs), adenomas with advanced histology, and cancers in an implementation study.

**Methods:**

We performed a secondary analysis of a large prospective colonoscopy cohort. All patients with SSLs, TSAs, advanced adenomas and/or cancers detected, and prospectively documented CADx-assisted or unassisted optical diagnoses were included. The primary outcome was the sensitivity of CADx-assisted and unassisted optical diagnoses for SSL identification. Secondary outcomes included diagnostic performance metrics for SSL, TSA, advanced adenoma, cancer identification and the impact of CADx output on endoscopists’ optical diagnoses.

**Results:**

In 2622 patients with 2456 eligible polyps, there were 140(5.7%) SSLs, 22(0.9%) TSAs, 213(8.7%) advanced adenomas, and 13(0.5%) cancers. Sensitivity for SSL identification of CADx-assisted and unassisted optical diagnosis was 55.7% (95%CI 42.9-67.8) and 38.6% (95%CI 22.7-57.3) respectively; p = 0.12 (**Table 1**). In CADx-assisted cases, endoscopists correctly identified SSLs more frequently when CADx classified these as non-neoplastic (56/79 cases) compared to neoplastic (23/79 cases),(66.7%[95%CI 51.8-78.9] vs 24.5%[95%CI 10.8-46.3]; p < 0.001), **Figure 1**. Sensitivity for TSA identification as neoplastic by CADx output was 61.6%(8/13), but CADx-assisted and unassisted endoscopist optical diagnosis identified TSAs as neoplastic in 91.1% and 77.8% of cases respectively.

**Conclusions:**

CADx use did neither harm nor improve optical diagnostic performance in polyps with SSL, TSA, advanced adenoma, or cancerous histology despite CADx inability to adequately classify these lesions, supporting safety but highlighting a key limitation of current CADx systems. Sensitivity for identification of these lesions trended universally higher with CADx-assisted optical diagnosis, though both CADx-assisted and unassisted ability to recognize SSLs, TSAs, and cancerous lesions remained low.

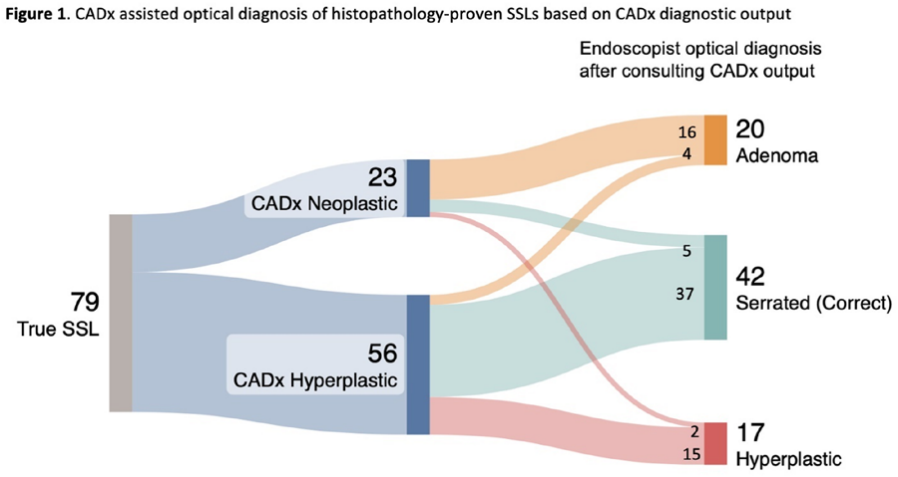

**Funding Agencies:**

CIHRTRIANGLE

**Table:** 

	SSL		TSA		Advanced Adenoma		Cancer	
	CADx assisted	Unassisted	CADx assisted	Unassisted	CADx assisted	Unassisted	CADx assisted	Unassisted
Polyps, n	79	37	12	9	71	83	6	7
Sensitivity	55.7 (42.9-67.8)	38.6 (22.7-57.3)	91.1 (52.3-99.0)	77.8 (37.6-95.0)	100.0 (100-100)	96.4 (89.1-98.9)	50.0 (14.2-85.8)	42.9 (12.3-80.1)
Specificity	88.3 (85.7-90.4)	94.0 (91.8-95.5)	-	-	-	-	99.8 (99.2-100)	99.7 (99.1-99.9)
Positive Predictive Value	25.9 (19.1-34.1)	19.9 (11.8-31.6)	-	-	-	-	60.0 (16.0-92.2)	50.0 (13.6-86.4)
Negative Predictive Value	96.0 (94.1-97.3)	97.9 (96.6-98.7)	99.8 (99.2-100)	99.7 (99.1-99.9)	100 (100-100)	99.7 (99.1-99.9)	99.7 (99.1-99.9)	99.6 (99.0-99.9)

* Optical diagnoses considered correct are the following: Serrated for SSLs, Serrated or Adenoma for TSAs, Adenoma for advanced adenomas, and Cancer for cancers, respectively

